# Pseudo-inflammatory manifestations of choroidal lymphoma resembling Vogt-Koyanagi-Harada disease: case report based on multimodal imaging

**DOI:** 10.1186/s12886-020-01353-9

**Published:** 2020-03-10

**Authors:** Kanae Fukutsu, Kenichi Namba, Daiju Iwata, Kazuomi Mizuuchi, Satoru Kase, Kayo Suzuki, Hiroshi Shimizu, Yukiko Shibata, Fumihiko Yamawaki, Masahiro Onozawa, Susumu Ishida

**Affiliations:** 1 Department of Ophthalmology, Faculty of Medicine and Graduate School of Medicine, Hokkaido University, N-15, W-7, Kita-ku, Sapporo, 060-8638 Japan; 2 Department of Hematology, Faculty of Medicine and Graduate School of Medicine, Hokkaido University, Sapporo, Japan

**Keywords:** Choroidal lymphoma, Indocyanine green angiography, Laser speckle flowgraphy, Serous retinal detachment, Vogt-Koyanagi-Harada disease

## Abstract

**Background:**

Hematologic malignancies occasionally cause serous retinal detachment (SRD); however, its pathogenesis remains unclear. Here we present the imaging characteristics of metastatic choroidal lymphoma masquerading as Vogt-Koyanagi-Harada (VKH) disease.

**Case presentation:**

A 45-year-old Japanese woman was referred to our clinic because of bilateral SRD with blurred vision. Fluorescein angiography revealed multiple pinpoint leakage followed by pooling OU. Enhanced depth imaging optical coherence tomography showed marked choroidal thickening OU. Laser speckle flowgraphy detected choroidal circulation impairment OU. Although these results totally agreed with the inflammatory manifestations of acute VKH disease, indocyanine green angiography demonstrated various sizes of sharply marginated hypofluorescent lesions that seemed atypical for the finding of VKH disease, i.e., vaguely marginated hypofluorescent small dots. Cerebrospinal fluid pleocytosis was not detected. Blood tests revealed leukocytosis together with elevation of lactate dehydrogenase and soluble interleukin-2 receptor levels. Corticosteroid pulse therapy did not improve any ocular findings. Bone marrow biopsy was then performed, leading to a definite diagnosis of diffuse large B-cell lymphoma. After starting systemic chemotherapy, both SRD and choroidal thickening resolved rapidly with visual recovery. However, choroidal hypoperfusion persisted, which contrasted distinctly with the inflammatory pattern of VKH disease, i.e., the restoration of choroidal blood flow in parallel with normalization of choroidal thickness.

**Conclusions:**

Our detailed multimodal observations highlighted the differential imaging features of choroidal lymphoma despite close resemblance to VKH disease especially at the initial stage. Impaired circulation in the thickened choroid marked the pseudo-inflammatory pathogenesis of SRD due to choroidal involvement with neoplastic, but not inflammatory cells.

## Background

Hematologic malignancies can involve every part of the eye, occasionally causing serous retinal detachment (SRD) [1, 2]. There are several case reports of a patient with systemic lymphoma or leukemia complicated by bilateral SRD simulating Vogt-Koyanagi-Harada (VKH) disease [3–7]. However, the pathogenesis of VKH disease-like ocular manifestations in hematologic malignancies remains to be clarified.

VKH disease, a systemic autoimmune disorder targeting melanocytes, induces inflammatory changes in melanocyte-associated multiple organs, such as uveitis, meningitis, hearing loss and skin depigmentation, and usually requires systemic corticosteroid therapy [8]. Uveitis (choroiditis) at the acute stage of VKH disease is associated with choroidal thickening [8] and impaired circulation [9], representing the inflammatory swelling of the choroid as well as the predisposing etiology of bilateral SRD.

Nearly half of patients with choroidal lymphoma, whether primary or secondary (metastatic), were reported to develop SRD [1]; however, treatment strategy is completely different between systemic lymphoma and VKH disease, underscoring the critical importance of differential diagnosis. To the best of our knowledge, this report is the first to show the multimodal imaging characteristics of choroidal lymphoma masquerading as VKH disease, dissecting the underlying pathogenesis of lymphoma-associated SRD.

## Case presentation

A 45-year-old Japanese woman was referred to our clinic because she developed bilateral SRD with blurred vision. The patient experienced occasional tinnitus after high fever had subsided several weeks before the initial visit to our hospital. Her visual acuity was 20/70 OD and 20/20 OS with mild myopia OU, and her intraocular pressure was normal OU. Slit-lamp microscopy did not detect any inflammatory findings, including iris nodules, keratic precipitates and anterior vitreous cells, except for the presence of occasional anterior chamber cells OU.

**Fig. 1:**
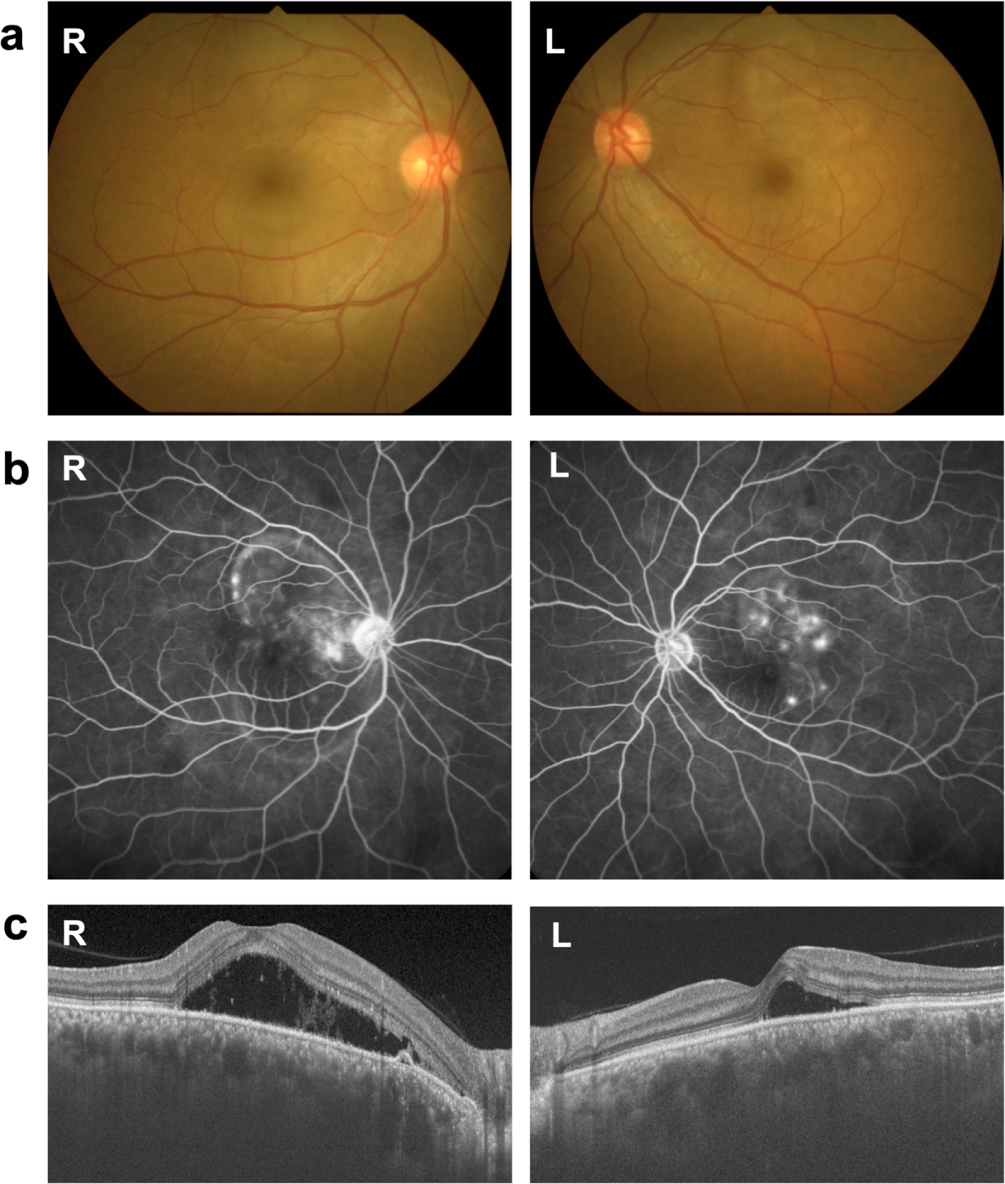
Fundus photographs, fluorescein angiography (FA), and enhanced depth imaging optical coherence tomography (EDI-OCT) at the first visit. Fundus photos showed bilateral serous retinal detachment (SRD) and optic disc swelling (**a**). FA detected multiple pinpoint leakage followed by pooling of the dye as well as optic disc staining in both eyes (**b**). EDI-OCT demonstrated SRD and marked choroidal thickening in both eyes (**c**). R: right, L: left

**Fig. 2:**
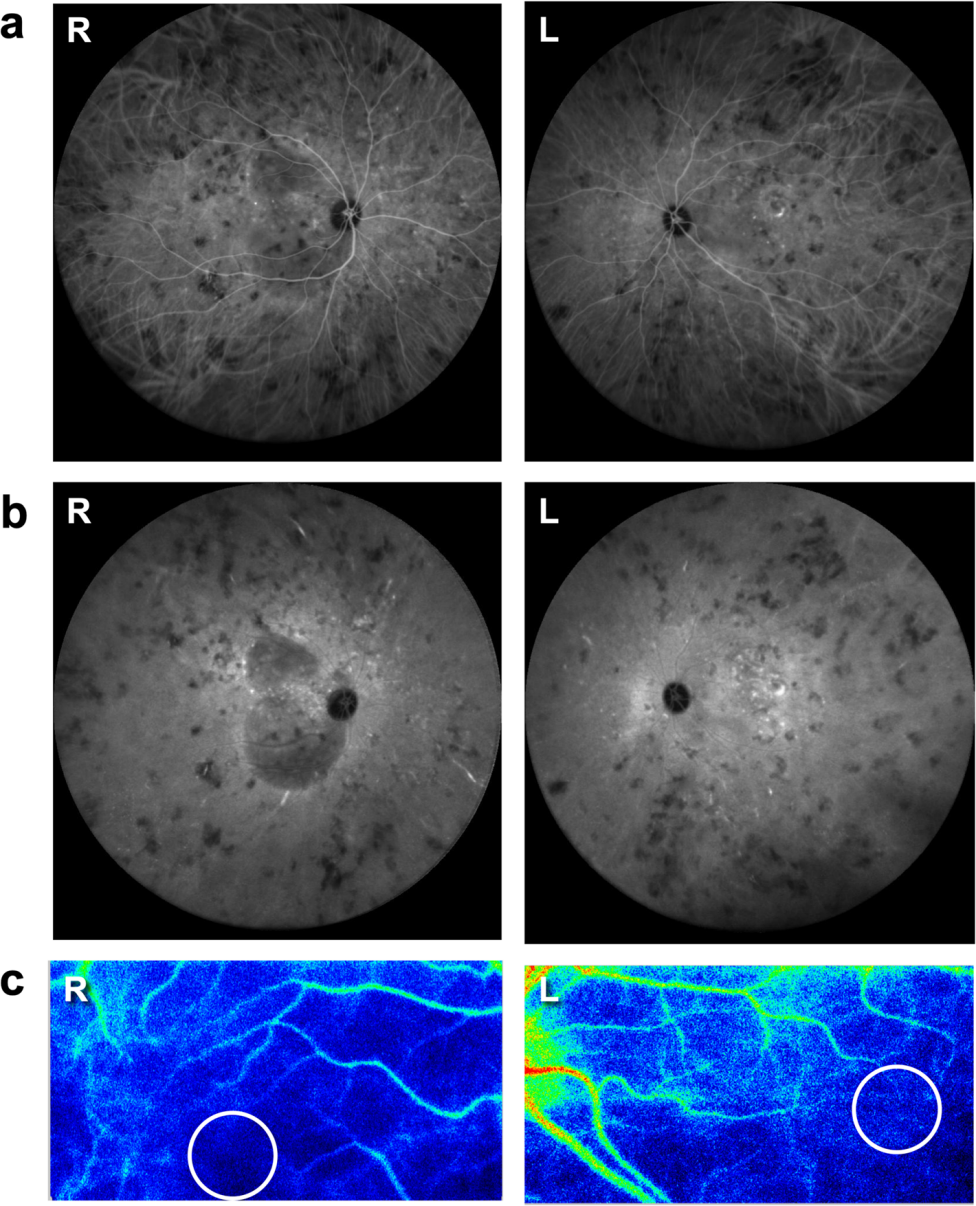
Indocyanine green angiography (ICGA) and laser speckle flowgraphy (LSFG) at the first visit. ICGA detected fuzzy vascular pattern of large stromal vessels in the mid-venous phase (**a**) and sharply marginated hypofluorescent lesions of various sizes that were clearly observed throughout the mid-venous phase (**a**) and the late phase (**b**). LSFG showed a cold-color pattern of the color map in the macular area bilaterally, and the mean blur rate (MBR) values in the circle were 2.16 in the right eye (R) and 2.63 in the left eye (L) (**c**). R: right, L: left

Fundus examination showed bilateral SRD and mild optic disc swelling but no vitreous opacity OU (Fig. 1a). Fluorescein angiography (FA) revealed multiple pinpoint leakage, dye pooling and optic disc staining OU (Fig. 1b). Enhanced depth imaging optical coherence tomography (EDI-OCT) demonstrated SRD and markedly thickened choroid filled with hyper-reflective interstitial materials OU (Fig. 1c). Indocyanine green angiography (ICGA) detected a fuzzy choroidal vascular pattern of large stromal vessels in the mid-venous phase OU (Fig. 2a), which was a consistent finding with typical VKH disease. However, sharply marginated hypofluorescent lesions in various sizes were clearly observed throughout the mid-venous and late phases OU (Fig. 2a, b), which seemed to be atypical for the finding of VKH disease, i.e., vaguely marginated hypofluorescent small dots. Laser speckle flowgraphy (LSFG) showed a cold-color (hypoperfused) pattern in the macular area OU (Fig. 2c), which agreed with the typical finding of VKH disease at the acute stage [9] .

Lumber puncture detected 4 cells/μL in the cerebrospinal fluid, indicating the absence of pleocytosis. Routine blood test yielded no apparent abnormal findings, except for lactate dehydrogenase (LDH) level (2645 U/L). Although the results of ICGA and lumber puncture were not suggestive of VKH disease as the causative etiology of bilateral SRD, the combined findings of all the other imaging modalities (FA, EDI-OCT and LSFG) were totally compatible with the inflammatory manifestations of acute VKH disease. The patient was started on oral prednisolone (PSL) at 40 mg/day; however, these ocular findings were unchanged for 7 days. The patient was then administered corticosteroid pulse therapy with 1000 mg of methylprednisolone for 3 days followed by 60 mg/day of PSL, which also failed to improve her ocular symptoms.

During the treatment, the patient complained of severe pain in her left knee and lower back, leading us to perform additional blood sampling that newly revealed marked elevation in LDH (4702 U/L) and soluble interleukin (IL)-2 receptor (3814 U/mL) levels together with leukocytosis (18,100/μL). Because the patient was suspected of having a hematological malignancy, she was referred to the hematology department of our hospital. Positron emission tomography-computed tomography detected ^18^F-fluorodeoxy glucose accumulation in her bones, pancreas, and lymph nodes. Bone marrow biopsy and subsequent histopathological and immunohistochemical analyses led to a definite diagnosis of CD5-positive diffuse large B-cell lymphoma (DLBCL).

The patient underwent 8 courses of chemotherapy, including 1 course of rituximab plus cyclophosphamide, doxorubicin, vincristine and PSL, 5 courses of rituximab with dose-adjusted etoposide, cyclophosphamide, doxorubicin, vincristine and PSL, 2 courses of high-dose methotrexate. The series of chemotherapy achieved complete remission of her systemic lymphoma as well as rapid resolution of SRD and choroidal thickening back to the normal range, concurrently with improvement of visual acuity to 20/16 OU.

**Fig. 3:**
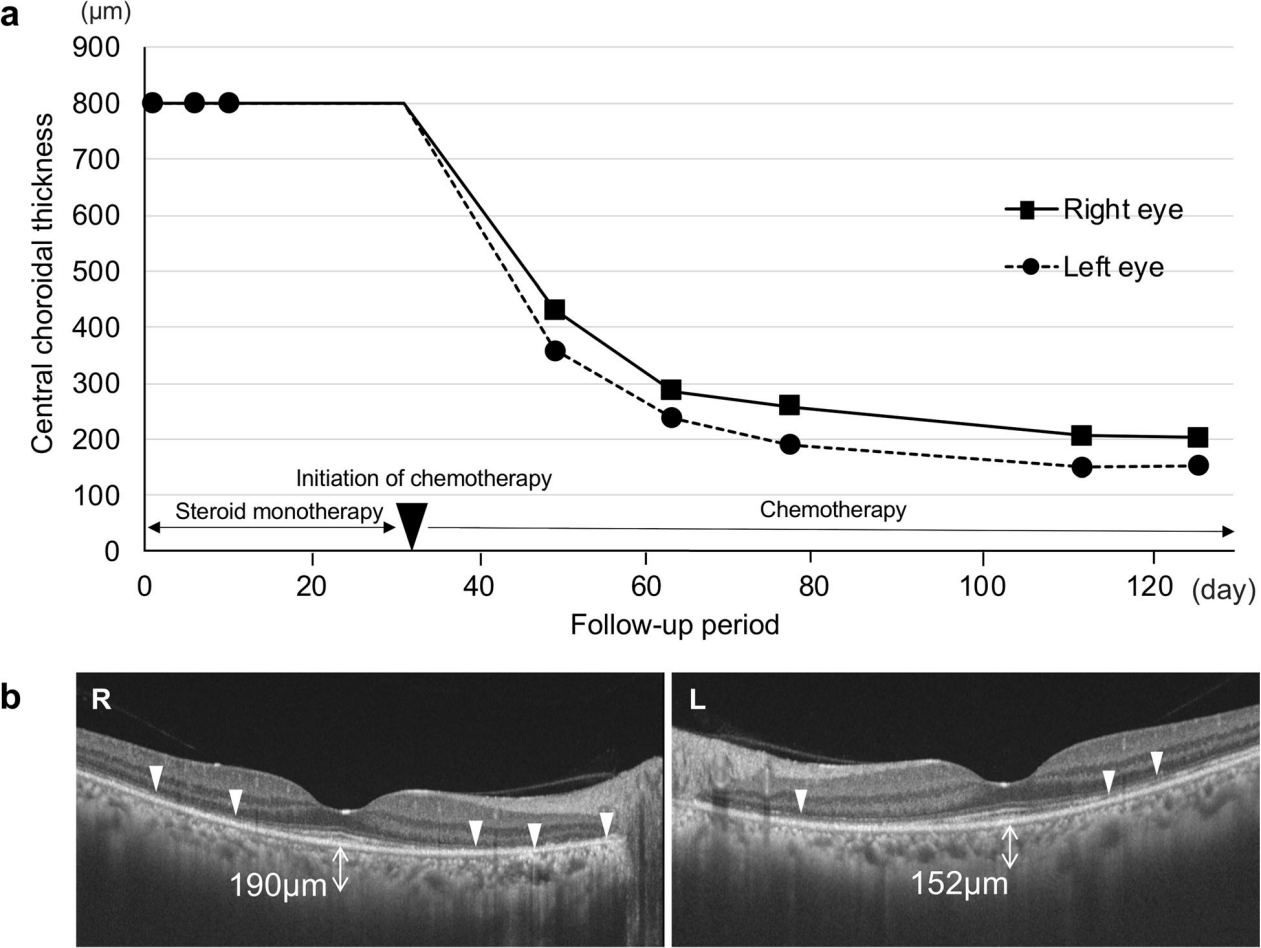
Change of choroidal thickness during the clinical course. EDI-OCT revealed that the thickened choroid was not changed with the corticosteroid treatment and was recovered rapidly along with the chemotherapy to the normal range (**a**). Moreover, EDI-OCT detected excessive thinning of the choroid beyond the normal range (190 μm in the right eye and 152 μm in the left eye) and extensive impairment of the outer retinal layer (white arrowheads) on day 126 (**b**)

**Fig. 4:**
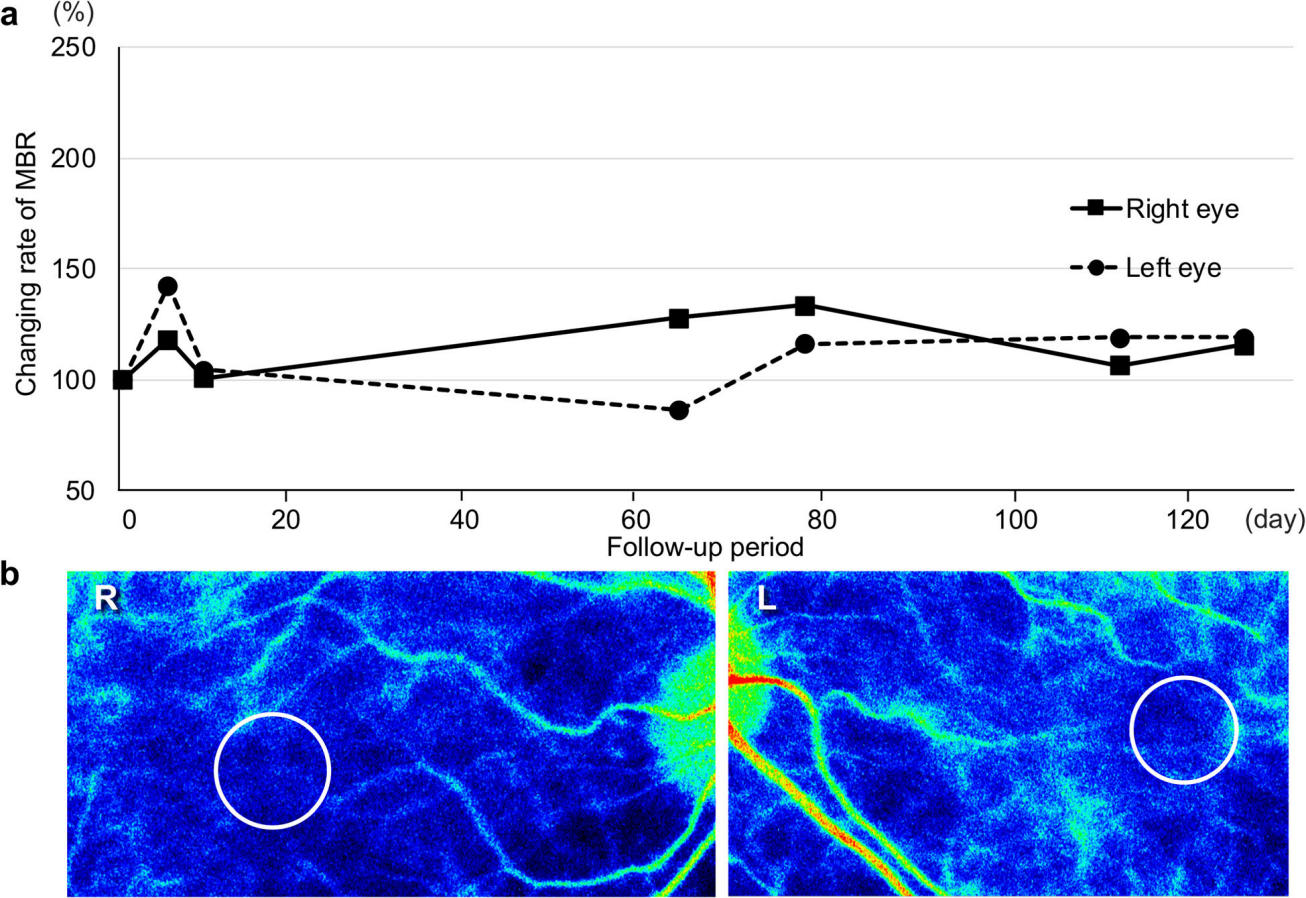
Change of choroidal circulation during the clinical course. LSFG revealed that the MBR remained low throughout the clinical course (**a**). Color map still showed a cold-color pattern, and the MBR values in the circle was 2.50 in the right eye (R) and 3.12 in the left eye (L) on day 126 (**b**)

However, EDI-OCT performed in the next few months detected progressive thinning of the choroid beyond the normal range and impairment of the outer retina (ellipsoid and interdigitation zones) (Fig. 3a, b), along with reduction in visual acuity (OU). Choroidal hypoperfusion in the macular area on LSFG persisted without any improvement of mean blur rate (MBR) values, the relative index of blood flow velocity, during the follow-up period (Fig. 4a, b). No major difference in ocular perfusion pressure values was observed throughout the entire course. The combination of EDI-OCT and LSFG findings after treatment contrasted distinctly with the inflammatory pattern of acute VKH disease, i.e., the restoration of choroidal circulation in parallel with normalization of choroidal thickness [9].

## Discussion and conclusion

The present case with bilateral SRD was initially suspected of suffering VKH disease due to the multimodal imaging characteristics based on FA, EDI-OCT and LSFG, all of which totally corresponded with the typical findings of acute VKH disease. However, the results of ICGA and lumber puncture, both of which were not suggestive of VKH disease, led us to consider the possibility of other diseases. The idea was also supported by non-responsiveness to systemic corticosteroid therapy and marked elevation of LDH and soluble IL-2 receptor. Finally, histopathological examination of the patient’s bone marrow resulted in the definite diagnosis of systemic DLBCL. The prompt improvement of SRD and choroidal thickening following chemotherapy confirmed choroidal involvement of DLBCL, the major cause of metastatic choroidal lymphoma [1], as the background etiology of the initial ocular manifestations resembling VKH disease. There are several case reports of a patient with systemic lymphoma or leukemia masquerading as VKH disease characterized by bilateral SRD [3–7]; however, the pathogenesis of hematologic malignancy-associated SRD remains unclear due to lack of detailed investigation using multiple imaging modalities.

EDI-OCT detected bilateral SRD and marked choroidal thickening, which are commonly observed in patients with VKH disease; particularly, marked choroidal thickening of more than 800 μm is a highly specific finding in VKH disease [10]. When systemic lymphoma involves the choroid, SRD is seen in almost 50% of patients, and is generally shallow and limited in extent [1]. In the present case, however, SRD was relatively bullous and extensive, which was similar to the findings of typical VKH disease. Furthermore, the detailed structure of the thickened choroid packed with hyper-reflective interstitial materials was consistent with inflammatory swelling of the choroid with massive infiltration of leukocytes, which is a hallmark of VKH disease at the acute stage. The definite diagnosis of DLBCL despite close resemblance to VKH disease in this patient suggests that EDI-OCT is not a reliable tool for differentiating between these two clinical entities featuring choroidal infiltration of metastatic lymphoma cells or autoimmune inflammatory lymphocytes.

In contrast, ICGA revealed partially different images in this patient as compared with typical VKH disease. The fuzzy vascular pattern of choroidal large vessels was observed in the mid-venous phase, which is also seen at the acute stage of VKH disease [11]. However, ICGA in our patient demonstrated various sizes of sharply marginated hypofluorescent lesions in the mid-venous to late phases, which seemed atypical for the finding of VKH disease, i.e., evenly sized blurred hypofluorescent dots that appear in the intermediate phase but become isofluorescent in the late phase [11]. Among multimodal imaging characteristics obtained at the initial stage, the detailed ICGA observation was the only way to consider further workup for a differential diagnosis rather than VKH disease, underscoring the importance of ICGA that is originally suitable for detecting choroidal abnormalities.

LSFG is a non-invasive technique to quantitatively measure MBR values, the relative index of blood flow velocity [12, 13], and useful for the evaluation of mainly deep choroidal vessels in the macular area [14]. We previously found that MBR increased significantly over time during systemic corticosteroid therapy in patients with primary or recurrent VKH disease [9, 15], suggesting that choroidal circulation was disturbed by infiltration of inflammatory leukocytes into the choroid. In our DLBCL patient, although bilateral SRD and choroidal thickening improved rapidly after the administration of chemotherapy, MBR remained low throughout the clinical course. The non-responsiveness of choroidal circulation in this patient is entirely different from the post-treatment flow restoration typically seen in VKH disease, suggesting that choroidal infiltration of neoplastic but not inflammatory cells caused persistent and irreversible damage to the vasculature. In accordance with the progressive thinning of the choroid currently seen on EDI-OCT, metastatic lymphoid cells are prone to bring tissue destruction presumably due in part to the proteolytic activity of matrix metalloproteinases [16–18].

Basically, causative mechanisms in choroidal thickening are largely divided into inflammatory and non-inflammatory etiologies, the latter of which would correspond to the pathogenesis of central serous chorioretinopathy (CSC). Importantly, a series of our recent data on LSFG has depicted the entirely opposite direction of blood flow changes in the choroid between inflammatory and non-inflammatory diseases. CSC is characterized by hyperperfusion of the thick choroid (also known as pachychoroid) [19–21], whereas circulatory disturbance (hypoperfusion) in the swollen choroid is a hallmark of various inflammatory diseases including acute posterior multifocal placoid pigment epitheliopathy [22], serpiginous choroiditis [23], unilateral acute idiopathic maculopathy [24], and punctate inner choroidopathy [25], on top of VKH disease [9, 15]. These inflammatory diseases are theorized to be associated with choroidal infiltration of leukocytes causing flow obstruction due to adhesion of cells to the inner wall of vessels followed by compression from outside of vessels surrounded with extravasated cells. We have recently reported that hypertensive chorioretinopathy shows a circulatory hemodynamics similar to CSC [26]. The present case showed a low value of MBR from the onset, which was contrary to the non-inflammatory, hyperperfused pattern seen in CSC and hypertensive chorioretinopathy.

The thickened choroid with impaired circulation, the inflammatory pattern on OCT and LSFG, was also observed in our recently reported case of chronic myeloid leukemia complicated by leukemic retinopathy, and termed the pseudo-inflammatory pattern [27]. The present case of DLBCL would be another example of pseudo-inflammatory manifestations of the choroid containing neoplastic but not inflammatory cells, suggesting close resemblance between hematologic malignancies and inflammatory diseases in terms of choroidal involvement especially at the early stage.

In summary, we reported a case of choroidal lymphoma with bilateral SRD and augmented choroidal thickness masquerading as VKH disease. Our detailed multimodal observations highlighted the differential imaging features of choroidal metastasis despite close resemblance to VKH disease. Impaired circulation in the thickened choroid marked the pseudo-inflammatory pathogenesis of SRD due to choroidal infiltration of lymphoma cells.

## Data Availability

Data sharing is not applicable to this article as no datasets were generated or analysed during the current study.

## Abbreviations

DLBCL: Diffuse large B-cell lymphoma
EDI-OCT: Enhanced depth imaging optical coherence tomography
FA: Fluorescein angiography
ICGA: Indocyanine green angiography
IL: Interleukin
LDH: Lactate dehydrogenase
LSFG: Laser speckle flowgraphy
MBR: Mean blur rate
PSL: Prednisolone
SRD: Serous retinal detachment
VKH: Vogt-Koyanagi-Harada
